# Human health in peril: The need to upgrade medical education in light of COVID-19

**DOI:** 10.3389/fmed.2022.999671

**Published:** 2022-10-03

**Authors:** Lukas Gatterer, Fabian Kriwan, Derrick Tanous, Katharina Wirnitzer

**Affiliations:** ^1^Department of Internal Medicine, Clinical Division of Internal Medicine III, St. Pölten University Hospital, Karl Landsteiner University of Health Sciences, St. Pölten, Austria; ^2^Department of Internal Medicine, Clinical Division of Internal Medicine I, State Hospital Feldkirch, Feldkirch, Austria; ^3^Department of Sport Science, Leopold-Franzens University Innsbruck, Innsbruck, Austria; ^4^Department of Research and Development in Teacher Education, University College of Teacher Education Tyrol, Innsbruck, Austria; ^5^Research Center Medical Humanities, Leopold-Franzens University Innsbruck, Innsbruck, Austria

**Keywords:** medical education, public health, nutrition, plant-based, NCD

## Abstract

While we might leave the COVID-19 pandemic behind, future health professionals are still confronted with another global phenomenon: the increasing pandemic of non-communicable diseases (NCDs). Both issues are strongly interwoven, yet current medical education fails to address their syndemic nature accordingly. There is scientific consensus that (i) most emerging infectious diseases are zoonotic, (ii) the overexploitation of earth's resources for animal protein production (i.e., tropical deforestation) rapidly escalates human contact with unknown pathogens, and (iii) people following a healthy plant-based diet present fewer rates of NCDs as well as severe illness and mortality from COVID-19. A shift toward whole food plant-based nutrition in the general population thus holds the potential to tackle both public health threats. We are convinced that it is every physician's responsibility to care for individual, public, and global health issues; however, future health professionals are not trained and educated regarding the health potential of plants and plant-based diets. The COVID-19 pandemic has demonstrated the urgent need for a “prevention first” approach. Therefore, in order to upgrade medical education worldwide and protect current and future human health properly, greater medical professional awareness of evidence on plant-based diets is urgently needed in classes, universities, and hospitals.

The COVID-19 pandemic has rocked the world, and people are still in survival mode. However, any crisis includes the potential to learn and grow. COVID-19 times have granted the opportunity to recapitulate and ask ourselves, as medical/public health experts and individuals, the questions too often left out of medical education; how many COVID-19 related deaths could have been prevented? And–what are the triggers of pandemics? Since this is not the first and certainly not the last pandemic (1).

Pandemic prevention is a major concern of global health. Experts warned for decades how anthropogenic exploitation of planet earth is driving risk (2). Medical students as future health professionals need to know about preventable aspects putting human health at peril. Medical schools and the WHO teach that 75% of emerging infectious diseases from the last decade are zoonotic (3). Ebola, the Zika virus, and bird flu, to name a few, transmitted from animals to humans (4), and SARS-CoV2 likely originates from animals, too (5). However, medical schools do not teach the fact that the destruction of natural habitats at an unprecedented pace recklessly exposes humankind to new diseases. Pointedly, the overexploitation of earth's resources for animal protein, including tropical deforestation, rapidly escalates human contact with unknown pathogens. Indeed, it is well-established that intensive livestock farming poses an imminent pandemic threat (4).

Like others (6), we are convinced that it is every physician's responsibility to care for individual, public, and global health issues. As medical professionals, we seem to have endorsed that people get sick as they get older (7). We may leave the COVID-19 pandemic behind, but the risk for future pandemics through the underlying pathology remains: preventable non-communicable diseases (NCDs), which are tightly linked to lifestyle (8). The major question to a young generation of future doctors remains: How can the physician professional group aid in tackling zoonotic and NCDs from the roots if the underlying mechanisms are not taught at medical schools? The time to talk about nutrition and its potential to harm or heal is now (9), especially with the rising demand for animal protein and its role in health (4).

In medical education, the benefits of a diet rich in fiber, fruits, and whole grains, while forgoing most animal products, saturated fat, and cholesterol are lightly introduced (10); limiting nutrition is the standard education medical students currently get from schools globally, rather than training healthy lifestyle behaviors (especially nutrition) as effective medical tools (11). The wealth of research regarding plant-based diets is astonishing to future doctors independently literature searching. Unfortunately, medical schools preclude education and training on the tremendous potential of “food as medicine,” which is still mostly untapped, including plant-based diets in preventing, treating, and even reversing some diseases [e.g., type 2 diabetes mellitus (12), coronary artery disease (13, 14), cancer (15)]. The Academy of Nutrition and Dietetics even states that well-planned plant-based diets provide several health benefits and are adequate for all stages of life (16), yet most doctors have never heard of it (17). All the more, medical schools often turn a blind eye to the health-threatening potential of specific foods, especially the conclusive evidence on meat in the development of some cancers (18, 19). As of 2015, the WHO classified processed meat as a class 1 carcinogen (18), yet most doctors do not advise against meat, and hospitals continue to serve it to patients, staff, and visitors. Finally, medical students are neither introduced to the sound body of evidence from large epidemiological and prospective cohort studies since 1978 indicating that cutting down on meat lowers NCD risk (20) nor to the fact that more premature deaths can be attributed to a suboptimal diet than tobacco smoking worldwide (8). Importantly, NCDs are the leading risk factors for COVID-19 infection and severe outcomes (21, 22).

In light of this pandemic–and the global, public, and individual health challenges we will face in the future–it is unacceptable for doctors to know so little about diet and nutrition. In a world plagued by NCDs, public health efforts should emphasize the focus of “prevention first” prescription in medical education, clinical settings, and health policymaking. Health education is often established by the state mandate of school curricula but is not seamless with tertiary curricula for future doctors and teachers, e. g. in Austria (23). Medical curricula worldwide should be standardized and taught with training in health-related knowledge and skills of powerful, evidence-based healthy lifestyle habits in order to create better individual and public health and protect humankind from the next pandemic. Therefore, as a self-evident part of the job description of medical experts, medical schools should teach and encourage students and patients to adopt a healthy lifestyle—evidence-based and up-to-date. Latest research shows that food as medicine is slowly but surely an issue to be addressed in standard medical education (24–26). A promising example is the Wayne State University School of Medicine, USA, where a mandatory 4-week plant-based nutrition curriculum educates medical students and clinicians on treating diet-related diseases (24). Also, a voluntary course on culinary medicine was persistently given positive feedback by attending students who enhanced their nutrition knowledge, particularly in counseling patients with diet-associated diseases, while improving their cooking skills (25). Similar programs must be implemented routinely within standardized medical education and training in order to enable future medical health professionals to give evidence-based consultation on clinical applications of plant-based nutrition after graduation.

To those in power, we urge you conclusively, not just as young doctors but as passionate and empathic inhabitants of this planet, to upgrade medical education and seize change the way modern medicine needs.

## Data availability statement

The original contributions presented in the study are included in the article/supplementary material, further inquiries can be directed to the corresponding author/s.

## Author contributions

This paper was conceptualized and written by FK and LG. The original draft was critically revised by DT and KW. The project was supervised by KW. All authors contributed to the article and approved the submitted version.

## Conflict of interest

The authors declare that the research was conducted in the absence of any commercial or financial relationships that could be construed as a potential conflict of interest.

## Publisher's note

All claims expressed in this article are solely those of the authors and do not necessarily represent those of their affiliated organizations, or those of the publisher, the editors and the reviewers. Any product that may be evaluated in this article, or claim that may be made by its manufacturer, is not guaranteed or endorsed by the publisher.

## References

[1] Madhav N Oppenheim B Gallivan M Mulembakani P Rubin E Wolfe N. Pandemics: risks, impacts, and mitigation. In: Jamison DT Gelband H Horton S., editors. Disease Control Priorities: Improving Health and Reducing Poverty. 3rd ed. Washington, DC: The International Bank for Reconstruction and Development / The World Bank (2017). 10.1596/978-1-4648-0527-1_ch17 Available online at: https://www.ncbi.nlm.nih.gov/books/NBK525302/ (assessed May 06, 2022).

[2] Report of the WHO/FAO/OIE joint consultation on emerging zoonotic diseases 3-5 May 2004-Geneva Switzerland. World Health Organization, Food and Agriculture Organization of the United Nations, World Organisation for Animal Health (2004). Available online at: https://apps.who.int/iris/bitstream/handle/10665/68899/WHO_CDS_CPE_ZFK_2004.9.pdf (assessed May 06, 2022).

[3] Report of the WHO EMRO Sixty-first session 19-22 October 2014-Tunis Tunisia. Zoonotic disease: emerging public health threats in the Region. (2014). Available online at: http://www.emro.who.int/fr/about-who/rc61/zoonotic-diseases.html (assessed May 07, 2022).

[4] UN. Preventing the next pandemic - Zoonotic diseases and how to break the chain of transmission. (2020). Available online at: https://www.unep.org/resources/report/preventing-future-zoonotic-disease-outbreaks-protecting-environment-animals-and (assessed May 06, 2022).

[5] Salata C Calistri A Parolin C Palù G. Coronaviruses: a paradigm of new emerging zoonotic diseases. Pathog Dis. (2019) 77:ftaa006. 10.1093/femspd/ftaa006 32065221 PMC7108526

[6] Moser AM Stigler FL Haditsch B. Physicians' responsibility for planetary health. Lancet Planet Health. (2017) 1:e56. 10.1016/S2542-5196(17)30023-2 29851580

[7] Tuso PJ Ismail MH Ha BP Bartolotto C. Nutritional update for physicians: plant-based diets. Perm J. (2013) 17:61–6. 10.7812/TPP/12-085 23704846 PMC3662288

[8] GBD 2017 Diet Collaborators. Health effects of dietary risks in 195 countries, 1990-2017: a systematic analysis for the Global Burden of Disease Study 2017. Lancet. (2019) 393:1958–72. 10.1016/S0140-6736(19)30041-8 30954305 PMC6899507

[9] The Lancet Editorial. We need to talk about meat. Lancet. (2018) 392:223. 10.1016/S0140-6736(18)32971-4 30496108

[10] Crowley J Ball L Hiddink GJ. Nutrition in medical education: a systematic review. Lancet Planet Health. (2019) 3:e379–89. 10.1016/S2542-5196(19)30171-8 31538623

[11] Trichopoulou A Orfanos P Norat T Bueno-de-Mesquita B Ocké MC Peeters PH. Modified Mediterranean diet and survival: EPIC-elderly prospective cohort study. BMJ. (2005) 330:991. 10.1136/bmj.38415.644155.8F 15820966 PMC557144

[12] Kahleova H Petersen KF Shulman GI. Effect of a low-fat vegan diet on body weight, insulin sensitivity, postprandial metabolism, and intramyocellular and hepatocellular lipid levels in overweight adults. A randomized clinical trial. JAMA Netw Open. (2020) 3:e2025454. 10.1001/jamanetworkopen.2020.25454 33252690 PMC7705596

[13] Esselstyn CB Ellis SG Medendorp SV Crowe TD. A strategy to arrest and reverse coronary artery disease: a 5-year longitudinal study of a single physician's practice. J Fam Pract. (1995) 41:560–8. 7500065

[14] Ornish D Scherwitz LW Billings JH Gould KL Merritt TA Sparler S. Intensive lifestyle changes for reversal of coronary heart disease. JAMA. (1998) 280:2001–7. 10.1001/jama.280.23.2001 9863851

[15] Ornish D Weidner G Fair WR Marlin R Pettengill EB Raisin CJ. Intensive lifestyle changes may affect the progression of prostate cancer. J Urol. (2005) 174:1065–9. Discussion 1069–70. 10.1097/01.ju.0000169487.49018.73 16094059

[16] Melina V Craig W Levin S. Position of the academy of nutrition and dietetics: vegetarian diets. J Acad Nutr Diet. (2016) 116:1970–80. 10.1016/j.jand.2016.09.025 27886704

[17] Abasi J. Medical students around the world poorly trained in nutrition. JAMA. (2019) 322:1852. 10.1001/jama.2019.17297 31670744

[18] International Agency for Research on Cancer. Volume 114: Consumption of red meat and processed meat. IARC Working Group. Lyon: IARC Monogr Eval Carcinog Risks Hum (2015). PMC7681469 1683674

[19] Aune D Navarro Rosenblatt DA Chan DSM Vieira AR Vieira AR Greenwood DC. Dairy products, calcium, and prostate cancer risk: a systematic review and meta-analysis of cohort studies. Am J Clin Nutr. (2015) 101:87–117. 10.3945/ajcn.113.067157 25527754

[20] Le LT Sabaté J. Beyond meatless, the health effects of vegan diets: findings from the Adventist cohorts. Nutrients. (2014) 6:2131–47. 10.3390/nu6062131 24871675 PMC4073139

[21] CDC. People with certain medical conditions. (2022). Available online at: https://www.cdc.gov/coronavirus/2019-ncov/need-extra-precautions/people-with-medical-conditions.html (assessed May 06, 2022).

[22] Horton R. Offline: COVID-19 is not a pandemic. Lancet. (2020) 396:874. 10.1016/S0140-6736(20)32000-6 32979964 PMC7515561

[23] Wirnitzer KC Drenowatz C. An integrative approach in addressing today's global health crisis. In: Wirnitzer K Drenowatz C Kirschner W Tanous D Rosemann T editors. International Research & Knowledge Exchange for Addressing Today's Global Health Paradox. 1st ed. Frontiers in Public Health (2020). p. 14–22. 10.3389/978-2-88966-537-2 Available online at: https://www.science2.school/wp-content/uploads/2021/05/International_Research_Knowledge_Exchange_for_Addressing_Todays_Global_Health_Paradox.pdf (accessed May 06, 2022).

[24] Mulpuri L Allen N Lunde A Thomas S Ray M Cret N. Rooting for wellness: an initiative introducing plant-based nutrition to first-year medical students. IJDRP. (2021) 3:120–31. 10.22230/ijdrp.2021v3n2a283

[25] Rothman JM Bilici N Mergler B Schumacher R Mataraza-Desmond T Booth M. A culinary medicine elective for clinically experienced medical students: a pilot study. J Altern Complement Med. (2020) 26:636–44. 10.1089/acm.2020.0063 32543207

[26] Morton KF Pantalos DC Ziegler C Patel PD. A place for plant-based nutrition in US medical school curriculum: a survey-based study. AJLM. (2022) 16:271–83. 10.1177/1559827620988677 35706597 PMC9189581

